# The role of disability and depressive symptoms in the relation between objective cognitive performance and subjective cognitive decline

**DOI:** 10.3389/fpsyt.2022.963703

**Published:** 2022-11-24

**Authors:** Deborah Pacifico, Serena Sabatini, Maddalena Fiordelli, Emiliano Albanese

**Affiliations:** Faculty of Biomedical Sciences, Institute of Public Health, Università della Svizzera Italiana, Lugano, Switzerland

**Keywords:** subjective cognitive complains, functional ability, mental health, depression, cognitive functioning

## Abstract

**Background:**

Subjective cognitive decline (SCD) and subjective memory decline (SMD) are common among older people. Evidence linking SCD and SMD with cognitive and memory impairment is inconsistent. Moreover, little is known about the associations of SCD and SMD with disability. We aimed to explore the associations of SCD and SMD with objective cognitive and memory performance, disability, and depressive symptoms.

**Materials and methods:**

In a cross-sectional study we conducted face to face interviews in a randomized sample of people aged ≥65 years living in the Canton of Ticino, southern Switzerland, between May 2021 and April 2022. We measured subjective cognitive decline with the MyCog, a subsection of the Subjective Cognitive Decline Questionnaire (SCD-Q); cognitive functioning with the Community Screening Instrument for Dementia; memory with the consortium to establish a registry for alzheimer’s disease (CERAD) 10-word list learning task; and disability and depressive symptoms with the world health organization disability assessment schedule 2.0 (WHO-DAS 2.0) and the Euro-Depression (EURO-D) scales, respectively.

**Results:**

Of the 250 participants 93.6% reported at least one cognitive difficulty, and 40.0% SMD. Both SCD and SMD were associated with poorer objective cognitive/memory performance, and independently with greater disability, and more depressive symptoms. But in participants with high disability and depressive symptoms subjective and objective cognition were no longer associated. Disability fully mediated the associations of poorer objective cognitive and memory performance with subjective cognitive and memory decline.

**Conclusion:**

Routine clinical assessments of cognitive function should include formal enquires about SCD and SMD, and also account for disability and depressive symptoms.

## Introduction

Up to one quarter of people aged 60 and over perceive a decline in their cognitive abilities, so-called subjective cognitive decline (SCD) and subjective memory decline, the self-reported experience of memory loss (SMD) (1). SCD is associated with worse cognitive performance, and with progressive impairment in cognitive function (2–5). Similarly, SMD appears to be associated with a higher risk of developing cognitive decline and with a worse memory performance (6–9). Therefore, the elicitation of SCD and SMD in routine clinical assessments of older adults may have important implications for timely detection of cognitive decline (10).

However, evidence on the associations between SCD and objective cognitive performance, and between SMD and objective memory performance is inconsistent. This may be ascribable to the lack of shared conceptualization and operationalization of SCD (11, 12), and to variations in the cognitive domains assessed: some studies focused on mnemonic tasks (13, 14), others explored several cognitive domains (15, 16). While the latter approach may be used to investigate non-amnestic forms of mild cognitive impairment (MCI) and dementia (17–22), memory impairment may be more noticeable, and the association between SMD and objective memory impairment appears to be stronger than the association of SCD and impairment across cognitive domains (2, 11).

Depressive symptoms are associated with both SCD and SMD in cognitively healthy individuals (23–31). Depressive symptoms are prospectively associated with cognitive impairment, and may be a prodromal sign of cognitive decline (7, 29–35). However, both objective and subjective decline in cognition, including memory may cause depressive symptoms (36–40).

Disability is defined according to the International Classification of Functioning, Disability, and Health (ICF) as a negative interaction between the individual health status and the environment, which results in functional difficulties. These difficulties may also be linked to SCD and SMD and cognitive impairment (41–44). Differently from instrumental activities of daily living (IADL), which focus on the practical consequence of one’s health state and may be spared in mildly cognitive impaired older adults, disability extends to a broad variety of potential consequences of poorer health status, and may capture subtle difficulties in a variety of lived experiences (41–43). And yet, the role that disability may play in the association of SCD and/or SMD with objective cognitive performance is largely unexplored (44, 45).

We aimed to study whether objective cognitive and memory performances were associated with subjective cognitive and memory decline, and with disability and depressive symptoms. We also explored the potential mediating or moderating effects of disability and depressive symptoms in the association of objective and subjective cognitive and memory functioning.

## Materials and Methods

### Study sample, design, and procedures

We used data from the SwissDEM Study^1^, a one-phase, population-based, cross-sectional study. In SwissDEM we recruited a randomized sample of the 80,500 people aged ≥65 years living in the Canton of Ticino, southern Switzerland (46), without any exclusion criteria except age. We sent an informative letter to 2,000 older adults randomly selected from local registries (response rate = 15%). Two weeks later, we sent an official invitation letter with instructions on how to participate in the study, including *via* a dedicated phone line, a paper-based form, and a direct web-link. All procedures and methodology for local adaptation, translation, piloting, and testing of all data collection tools, and for the interviewers’ training have been previously reported (47). Briefly, overall 24 study interviewers received standard instructions and training for the cognitive assessments by two neuropsychologists. The training was coordinated by the PI (EA) and followed the original 10/66 manual which was previously used in community settings (48), and was adapted to the study context and culture. The training included sessions covering the theoretical background of cognitive impairment and decline, detailed explanation of the cognitive assessment tools, practical group and individual activities on cognitive tests administration and answers coding, and all sampling, registration and data collection procedures.

We conducted all interviews face to face between May 2021 and April 2022 with participants (i.e., older adults) and informants (i.e., a close family member, friend, or who knew the participant well), in participants’ home or in dedicated areas in a local older adults’ association, and in our lab at Università della Svizzerza italiana (in Lugano). We used RedCap (i.e., Research Electronic Data Capture) on dedicated tablets with data encryption for both online and off line data collection, which also allowed automated and regular checks, and monitoring of data collection standards and procedures throughout data collection (47).

### Ethical approval

All participants signed a paper-based informed consent to participate in the study, which was authorized by the local Ethics Committee (ID 2017-02181).

### Measures

#### Sociodemographic information

Sociodemographic variables comprised age, sex, educational level, and marital status. Educational level comprised six categories: No education; Not completed primary school; Primary school; Secondary school; High school; University certificate. Marital status comprised five categories: Single; Married; Divorced/Separate; Widowed; Civil union.

#### Subjective cognitive decline

To assess subjective decline in memory, language, and executive functioning in the last two years we used the MyCog, a subsection of the Subjective Cognitive Decline Questionnaire (SCD-Q) (49). The MyCog SCD-Q scale comprises 24 dichotomous items (yes = 1; no = 0); higher scores (possible range: 0–24) indicate greater SCD. Sample items are “I find it harder to learn new telephone numbers” and “I find it harder to concentrate on what I am doing.” Study authors (DP; EA) translated this measure from English to Italian following standard procedures for translation and back translation (50). We conducted brief internal cognitive interviews and resolved discrepancies and incongruities through discussion among member of the research group (DP; EA; GF; BG). Cronbach’s alpha (α) for the SCD-Q in the current study sample is 0.68 indicating acceptable scale reliability.

#### Subjective memory decline

Subjective memory decline was determined with a positive answer to the bespoken question: “Do you perceive cognitive difficulties, such as memory problems?” Similar assessment tools have been used in the literature to investigate SMD (51, 52).

#### Objective cognitive performance

To assess objective cognitive performance we used the Community Screening Instrument for Dementia (CSI’D’) participant part (53). The CSI’D’ is a widely used, culturally unbiased and education-fair instrument for dementia screening. The test covers various domains comprising orientation, memory, language expression, comprehension, and spatial constructional praxis. The total score is calculated by summing the 35 scale items. Higher total score (possible range: 0–35) means better cognitive performance.

#### Objective memory performance

We used the consortium to establish a registry for alzheimer’s disease (CERAD) 10-word list learning task to assess memory. The test consists of a list-learning paradigm in which participants listen 10 words and are asked to recall as many as possible; the process is repeated three times (immediate recall). Around 5 min after participants are asked again to list all the words they remember (deferred recall). The total score for both immediate (possible score: 0–30) and deferred recall (possible score: 0–10) is obtained from the sum of correctly remembered words. The total CERAD 10-word list learning task score is obtained from the sum of immediate and deferred recall scores (possible range: 0–40). Higher scores mirror better performance (54).

#### Disability

The short version of the World Health Organization Disability Assessment Schedule 2.0 (WHO-DAS 2.0) was used to assess disability in the previous 30 days. Each of the 12 sel-reported items is scored on a Likert scale ranging between one (none) and five (extreme) (55). Sample items are “In the last month, how much difficulty did you have in carrying out your day to day work and usual activities?” and “In the last month, how much of a problem did you have joining community activities in the same way as anyone else can?” Higher total scores (possible range: 12–60) indicate more pervasive disability.

#### Depressive symptoms

We assessed depressive symptoms over the past month with the EURO-D. For each of the 12 items a score of 1 indicates that the selected symptom is present whereas a score of 0 indicates that the selected symptom is not present. A sample item is “In the last month, have you been sad or depressed?” Higher scores (possible range: 0–12) indicate greater depressive symptoms (56). The original version of the EURO-D includes an item assessing concentration problems, which might capture SCD. For the purpose of the current study, and consistent with previous approaches (57), we computed the EURO-D total score without the item assessing concentration problems (possible score range: 0–11).

#### Health conditions

We assessed participants’ health conditions through standard self-reported questions derived from the 10/66 protocol (58). Health conditions comprised stroke, ischemic heart disease, heart problems, hypertension, diabetes, episodes of loss of consciousness, chronic bad chest, arthritis, dyspnea, and gastrointestinal problems. We generated an overall score indicating the number of health conditions using a cumulative approach.

### Statistical analysis

We reported descriptive statistics for all study variables. We used chi2 tests and *t*-tests to examine differences in the scores of study variables between those included and those excluded from the current study analyses.

We used Pearson’s correlation and the point-biserial correlation in a correlation matrix to examine bivariate correlations among all study measures and covariates.

We conceived four regression models on *a priori* hypothesis. Previous evidence showed that SCD is associated with objective cognitive performance (2–5), disability (44), and depressive symptoms (23, 24); similarly, SMD is associated with objective memory performance (6, 7), disability (45), and depressive symptoms (27). However, evidence on these associations is inconsistent. Therefore, we fitted linear regression models to examine the associations of SCD (outcome) with objective cognitive performance, disability, and depressive symptoms (predictors), and of SMD (outcome) with objective memory performance, disability, and depressive symptoms (predictors). To explore within one model the relationship between the outcome and several predictors, we also fitted a multiple regression model including within the same model objective cognitive performance, disability, and depressive symptoms as predictors of SCD. Similarly, we fitted a multiple regression model including within the same model objective memory performance, disability, and depressive symptoms as predictors of SMD. For each regression we fitted both an unadjusted and an adjusted (for age, sex, and educational level) model, as previous evidence showed that SCD and SMD might be associated with age, gender, and education (59).

We used tests of interaction to examine whether levels of disability and depressive symptoms moderate the relationship between objective cognitive performance and SCD. Similarly, we used tests of interaction to examine whether levels of disability and depressive symptoms moderate the relationship between objective memory performance and SMD. When a test of interaction was statistically significant at the 5% level, we reported the unstandardized regression coefficients of the interaction terms, and the relationship between objective cognitive performance and SCD and/or between objective memory performance and SMD for three subgroups based on tertiles of the selected moderating variable. We then used the *sem* interface in STATA to fit mediation models to explore the mediating role of disability in the associations of objective cognitive and memory performance (predictors) with SCD and SMD (outcomes). We fitted both unadjusted and adjusted (for age, sex, and educational level) models.

To maximize use of available data, mean imputation and imputation of the most frequent value was used when a response for one of items of the WHO-DAS 2.0 was missing (this was done for two participants) and when a response for one of items of the EURO-D was missing (this was done for three participants).

We reported standardized regression coefficients (effects sizes) to quantify the associations; coefficients ≤0.09 were considered negligible, 0.10–0.29 small, 0.30–0.49 moderate, and ≥0.50 large (60). Analyses were conducted in STATA version 16 (61).

## Results

### Descriptive statistics

Descriptive statistics for the main study variables are reported in Table 1. After the exclusion of 49 participants because of missing data in the main outcome and exposure variables, the resulting analytic sample comprised 250 participants, none of which self-reported a previous diagnosis of dementia.

**TABLE 1:** Descriptive characteristics of the study sample.

	Total study sample (*n* = 250)
Age, M (SD; range)	75.9 (6.31; 65–92)
Women, *n* (%)	118 (47.2)
**Educational level, *n* (%)**	
No education	1 (0.4)
Not finished primary school	2 (0.8)
Primary school	18 (7.2)
Secondary school	51 (20.4)
High school	136 (54.4)
University/Professional certificate	41 (16.4)
Missing	1 (0.4)
**Marital status, *n* (%)**	
Single	19 (7.6)
Married	160 (64.0)
Divorced/separated	29 (11.6)
Widowed	39 (15.6)
Civil union	2 (0.8)
Missing	1 (0.4)
Disability, M (SD)	4.38 (5.98)
Depressive symptoms, M (SD)	2.10 (1.71)
Depressive symptoms without attention^a^, M (SD)	1.88 (1.59)
**Number of health conditions, *n* (%)**	
Below three	181 (72.6)
Three or more	69 (27.6)

^a^For the purpose of the current study, an alternative depressive symptoms score without the item assessing concentration problems was computed.

Participants’ mean age was 75.9 years (SD = 6.31). Slightly below half (47.2%) were women. Most participants completed at least secondary education (91.2%), and 16.4% had an academic degree. The majority were married (64.0%).

On average participants had intact cognitive functioning, as indicated by their means scores on the CSI’D’ (M = 31.66; SD = 2.58; Range: 21.32–35). The mean CERAD 10-word list learning task was 21.53 (SD = 6.14). Participants reported cognitive difficulties. Almost everyone (93.6% of the study sample) reported at least one cognitive difficulty in the SCD-Q and 40% reported SMD (yes/no). The mean on the WHODAS 2.0 disability was 4.38 (SD = 5.98). On average participants reported two depressive symptoms, and having received either one or two clinical diagnoses of health conditions (72.6%) (Supplementary Table 1). Table 2 shows the correlation matrix of study variables.

**TABLE 2:** Bivariate correlations of subjective cognitive and memory decline with objective cognitive and memory performance, depressive symptoms, disability, and participants’ age, sex, and educational levels.

	1	2	3	4	5	6	7	8	9
Subjective cognitive decline	1	–	–	–	–	–	–	–	–
Subjective memory decline	0.469**	1	–	–	–	–	–	–	–
Objective cognitive performance	−0.260**	−0.129*	1	–	–	–	–	–	–
Objective memory performance	−0.194**	−0.160*	0.470**	1	–	–	–	–	–
Depressive symptoms	0.233**	0.205**	−0.148*	–0.065	1	–	–	–	–
Disability	0.341**	0.276**	−0.411**	0.257**	0.368**	1	–	–	–
Age	0.362**	0.149*	−0.410**	−0.301**	0.134*	0.373**	1	–	–
Sex^a^	–0.091	–0.003	–0.016	0.69	0.172**	0.111	0.003	1	–
Educational level	–0.019	0.050	0.232**	0.184**	–0.072	–0.059	−0.133*	−0.165**	1

*0.05 level (two-tailed).

**0.01 level (two-tailed).

^a^point-biserial correlations between gender (1, male; 2, female) and other variables.

### Associations of subjective cognitive decline with objective cognitive performance, disability, and depressive symptoms

Associations of SCD with objective cognitive performance, disability, and depressive symptoms are reported in Table 3. In regression models adjusted for age, sex, and education (model 2) poorer objective cognitive performance (ß = –0.13; 95% CI:–0.25; –0.003), greater disability (ß = 0.26; 95% CI: 0.14; 0.37), more depressive symptoms (ß = 0.21; 95% CI: 0.10; 0.32) were all positively and significantly associated with higher scores of SCD. In the mutually adjusted model disability (ß = 0.19; 95% CI: 0.06; 0.32), and more depressive symptoms (ß = 0.15; 95% CI: 0.03; 0.26) remained significantly associated with SCD scores.

**TABLE 3:** Associations of subjective cognitive decline with objective cognitive performance, disability, and depressive symptoms.

	Predictors of subjective cognitive decline		
	Objective cognitive performance (COGSCORE)	Disability (WHODAS 2.0)	Depressive symptoms (EURO-D)
	ß (95% CI); *P-value*	ß (95% CI); *P-value*	ß (95% CI); *P-value*
Model 1	–0.23 (–0.35; –0.12); <0.001	0.34 (0.24; 0.45); <0.001	0.23 (0.12; 0.35); <0.001
Model 2	–0.13 (–0.25; –0.003); 0.045	0.26 (0.14; 0.37); <0.001	0.21 (0.10; 0.32); <0.001

Model 1, linear regression models showing the associations of SCD (outcome) with objective cognitive performance, disability, and depressive symptoms (predictors). Model 2, linear regression models showing the associations of SCD (outcome) with objective cognitive performance, disability, and depressive symptoms (predictors) and adjusting for age, sex, and educational level.

### Associations of subjective memory decline with objective memory performance, disability, and depressive symptoms

Associations of subjective memory decline (SMD) with objective memory performance, disability, and depressive symptoms are reported in Table 4. In regression models adjusted for age, sex, and education (model 2) better objective memory performance (OR = 0.95; 95% CI: 0.91; 1.0) predicted less likelihood of reporting SMD (yes/no). Conversely, greater disability (OR = 1.10; 95% CI = 1.04; 1.17), and more depressive symptoms (OR = 1.31; 95% CI: 1.10; 1.56) predicted higher likelihood of reporting SMD. In further mutually adjusted models, for both disability (OR = 1.08; 95% CI: 1.02; 1.14) and depressive symptoms (OR = 1.21; 95% CI: 1.01; 1.45) predicted the likelihood of reporting SMD.

**TABLE 4:** Associations of subjective memory decline with objective memory performance, disability, and depressive symptoms.

	Predictors of subjective memory decline		
	Objective memory performance (CERAD 10-word list learning task)	Disability (WHODAS 2.0)	Depressive symptoms (EURO-D)
			
	OR (95% CI); *p*-value	OR (95% CI); *p*-value	OR (95% CI); *p*-value
Model 1	0.95 (0.91; 0.99); 0.013	1.11 (1.05; 1.17); <0.001	1.31 (1.11; 1.54); 0.002
Model 2	0.95 (0.91; 1.0); 0.043	1.10 (1.04; 1.17); <0.001	1.31 (1.10; 1.56); 0.002

Model 1, logistic regression models showing the associations of SMD (outcome) with objective memory performance, disability, and depressive symptoms (predictors). Model 2, logistic regression models showing the associations of SMD (outcome) with objective memory performance, disability, and depressive symptoms (predictors) and adjusting for age, sex, and educational level.

### Moderating role of disability and depressive symptoms in the association of objective cognitive performance with subjective cognitive decline

The interaction term of disability and objective cognition was statistically significant in the association with SCD (ß = 0.01; 95% CI:0.01; 0.12; *p* < 0.001). The first third of disability comprised 106 participants who scored either 0 or 1 on the WHO-DAS 2.0. The second third comprised 66 participants who scored between 2 and 4 on the WHO-DAS 2.0. The upper third comprised 78 participants who scored between 5 and 39 on the WHO-DAS 2.0. In the stratified analysis, the association of objective cognition with SCD was not significant across disability (all *p*-values ≥ 0.075).

The interaction term of depressive symptoms and objective cognitive performance was a statistically significant predictor of SCD (ß = 0.02; 95% CI: 0.01; 0.03; *p* = 0.001). The lower third of depressive symptoms severity comprised 118 participants who scored either 0 or 1 on the EURO-D. The middle third comprised 53 participants who scored 2 on the EURO-D, and the higher third comprised 78 participants who scored between 3 and 8 on the EURO-D scale. In the stratified analysis, there was a significant association between objective cognitive function and SCD in those in the lower (ß = –0.31; 95% CI: –0.47; –0.15) and middle (ß = –0.27; 95% CI: –0.51; –0.03) tertile of depressive symptoms, but not among those in the higher tertile of depressive symptoms (*p* = 0.480). In the adjusted model the association between objective cognition and SCD remained significant only among those in the lower tertile of depressive symptoms (ß = –0.22; 95% CI: –0.39; –0.05).

### Moderating role of disability and depressive symptoms in the association of objective memory performance with subjective memory decline

Disability significantly modified the association between objective memory performance and SMD (Interaction term: ß = 0.01; 95% CI: 0.01; 0.02; *p*-value < 0.001). Those with higher objective memory performance were less likely to report SMD among those in the lower tertile of disability (OR = 0.91; 95% CI = 0.83; 1.00). This association was adjusted away by age, sex, and educational level (*p* = 0.971). The associations of objective memory performance and SMD were not significant in those in the middle (*p* = 0.439) and higher (*p* = 0.514) tertiles of levels of disability scores.

Depressive symptoms significantly modified the association of objective memory performance with SMD (Interaction term: ß = 0.02; 95% CI: 0.004; 0.04; *p* = 0.012). Those with better objective memory performance were less likely to report SMD if they had lower depressive symptoms (OR = 0.91; 95% CI = 0.84; 0.98), but not after adjustment (*p* = 0.094). Similarly, those with better objective memory performance were less likely to report SMD if they had intermediate EURO-D depressive symptomatology (OR = 0.90; 95% CI: 0.81;0.99), also after adjustment for age, sex, and education (OR = 0.89; 95% CI: 0.80; 0.99). We found no significant association between objective memory performance and SMD in those with highest EURO-D scores (*p* = 0.688).

### Mediating role of disability and depressive symptoms in the association of objective cognitive performance with subjective cognitive decline

**FIGURE 1:**
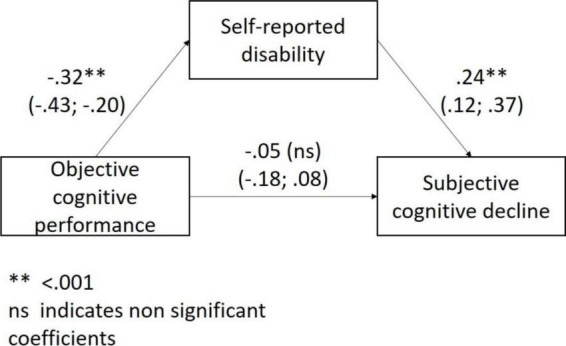
The mediating role of disability in the association between objective cognitive performance and subjective cognitive decline.

In the regression models, objective cognitive performance was significantly associated with disability (ß = –0.31; 95% CI: –0.42; –0.20) but not with depressive symptoms (*p* = 0.552). Hence, we only tested the mediating role of disability in the association of objective cognitive performance with SCD. Both in the unadjusted and adjusted (for age, sex, and education level) mediating model disability fully mediated the association of objective cognitive performance with SCD (Figure 1).

### Mediating role of disability and depressive symptoms in the association of objective memory performance with subjective memory decline

**FIGURE 2:**
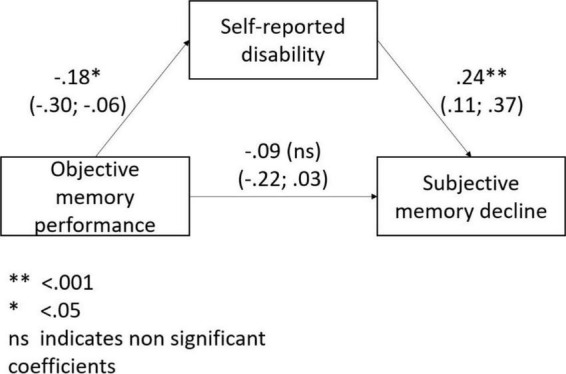
The mediating role of disability in the association between objective memory performance and subjective memory decline.

Objective memory performance was significantly associated with disability (ß = –0.18; 95% CI: –0.31; –0.06) but not with depressive symptoms (*p* = 0.129). Both in the unadjusted and adjusted (for age, sex, and education level) mediating model disability significantly mediated the association of objective memory performance with SMD (Figure 2).

## Discussion

In a sample of older adults (65 +) living in southern Switzerland (i.e., Ticino) both overall cognitive (SCD) and memory-specific subjective (SMD) complaints were common. SCD and SMD were associated with poorer objective cognitive/memory performance, and independently with disability, and depressive symptoms. Subjective and objective cognition were not associated in those with high disability and depressive symptoms. However, disability mediated the associations of objective cognitive and memory performance with subjective cognitive and memory decline. The significant associations we found between disability and SCD/SMD suggest that perceived difficulties in daily functioning may alter the subjects’ perception of their cognitive capabilities (62–64).

Our findings on the positive associations between disability and subjective cognitive and memory decline are novel. Other studies found that individuals experiencing SCD have more difficulties in daily activities (44), and worse physical function (45). In our study participants with higher disability were more likely to report SMD irrespective of their cognitive impairment. Nevertheless, IADL seem fairly preserved in older adults with subjective cognitive complaints (65). This may suggest a preserved independence despite difficulties in daily functioning.

The associations of objective cognitive and memory performances with subjective cognitive and memory decline were fully mediated by disability. Cross-sectional associations between greater cognitive impairment and greater disability assessed with the WHO-DAS 2.0 tool have been previously reported (66). It is plausible that the consequences of cognitive impairment on functional ability may contribute to the self-perception of cognitive deficits, as individuals experience limitations in their daily life (44).

The associations we found between more depressive symptoms and SCD and SMD are in line with a large corpus of evidence (23–31). Issues of directionality remain unresolved but the association may be bi-directional. Prospective studies suggest that depression is a risk factor for objective cognitive decline (7, 29–32, 66), and that cognitive impairment and neurodegenerative changes may cause depressive symptoms (21, 67, 68), and mood changes (32), respectively. Depressive symptoms and cognitive decline may also share common causal and precipitating factors such as vascular problems (69, 70).

Our findings on the lack of association between objective and subjective cognitive function in people with more marked depressive symptoms may be explained by the prominence of mood rather than cognitive concerns in these individuals. It might be that depressive symptoms entail a negative perspective on several aspects of individuals’ lives, including one’s cognitive capabilities (71). However, depression can be a consequence of both objective cognitive decline and SCD (24, 68, 72). Whether and the extent to which depressive symptoms moderate the associations of objective cognition with SCD in older people with cognition impairment warrants further investigations.

Some potential implications of our findings are worth noting. Because SCD and SMD may be a prodromal symptom of dementia (3) they could be elicited in routine clinical assessments to enhance timely diagnosis, and accounting for the disability level and depressive symptoms of the individual.

Our results suggest that people may not know what is expected and normative in cognitive functioning and might ascribe the perceived cognitive difficulties to dementia; this might be due to dementia worry among older people (68) or, conversely, support the erroneous belief that dementia is a normal part of aging (73). Awareness, knowledge, and understanding of dementia and cognitive decline, and their signs and symptoms must improve in the general public. Access to and use of services should be dictated by actual needs. Worry-well individuals may benefit from psychological help to address psycho-affective symptoms which might influence the perception they have of their cognitive capabilities.

This study has several strengths. First, objective cognitive performance was assessed with a comprehensive cognitive battery administered to the participant in person by trained interviewers (47). Moreover, our assessments were previously validated in the study region (Ticino) (47). Second, we assessed SCD using a comprehensive, reliable, and valid tool that covers perceived difficulties in several cognitive domains. Third, the MyCog SCD-Q returns a total score on a discrete rather than categorical scale (11), which allowed a better quantification of the severity of SCD (74). Fourth, our investigation of the moderating role of disability in the associations of objective cognitive performance with SCD, and of objective memory performance with SMD is novel. Finally, we conducted our study in community-dwelling individuals, and extended previous evidence from clinical settings. The representativeness of the target population of our study sample supports the external validity of our observations.

Some limitations are worth noting. First, the cross-sectional nature of the study leads to issues of directionality, which may diminish the interpretability also of the mediating models. Second, despite none of the study participants self-reported a previous diagnosis of dementia, we did not have access to medical records or clinical assessments; however, the minimum achieved score in the CSI’D instrument in our sample (MIN = 21.32) suggests that cognitive impairment was not severe in any of the study participants. We acknowledge that this may introduce some undue selection bias, though potentially non-differential with respect to both objective and subjective cognitive impairment. Third, although the WHO-DAS 2.0 is a robust and extensively validated disability scale, it relies on self-reporting rather than physical or functional assessments (i.e., objective indicators of disability). Fourth, in this study we did not rely on a medical diagnosis of depression, but the EURO-D scale is commonly used in population-based samples to assess depressive symptomatology.

## Conclusion

We reported on the cross-sectional associations of objective cognitive and memory performances, disability, and depression with subjective cognitive and memory decline in a population-based sample of older adults. Subjective complaints about cognition were common, signaling a high level of concern in older adults about their cognitive abilities in late life. Health policies should aim to improve awareness, understanding, and knowledge about dementia in the general population to improve and enhance access and use of diagnostic and care services, timely and appropriately. At the same time, enquires in routine clinical assessments about SCD and SMD can contribute to timely detection of cognitive decline, and should account for the potentially modifying and mediating effects of both disability and depressive symptoms.

## Data availability statement

The raw data supporting the conclusions of this article will be made available by the authors, without undue reservation.

## Ethics statement

The studies involving human participants were reviewed and approved by Ethics Committee of the Canton of Ticino. The patients/participants provided their written informed consent to participate in this study.

## Author contributions

DP contributed to the study design, data collection, and took the lead in writing the manuscript. SS conducted the data analysis, contributed to the writing, and reviewing of the manuscript. MF contributed to the study design, writing, and reviewing of the manuscript. EA served as PI of the SwissDEM study, designed the SwissDEM study, and reviewed the manuscript. All authors read and approved the final manuscript.

## Footnotes

1
https://www.biomed.usi.ch/en/facolta-scienze-biomediche/istituti/swissdem

## References

[1] Roehr S Pabst A Riedel-Heller SG Jessen F Turana Y Handajani YS Estimating prevalence of subjective cognitive decline in and across international cohort studies of aging: a COSMIC study. *Alzheimers Res Ther.* (2020) 12:167. 10.1101/2020.05.20.20106526 PMC7749505 33339532

[2] Jessen F Amariglio RE van Boxtel M Breteler M Ceccaldi M Chételat G A conceptual framework for research on subjective cognitive decline in preclinical Alzheimer’s disease. *Alzheimers Dement.* (2014) 10:844–52. 10.1016/j.jalz.2014.01.001 24798886 PMC4317324

[3] Amariglio RE Becker JA Carmasin J Wadsworth LP Lorius N Sullivan C Subjective cognitive complaints and amyloid burden in cognitively normal older individuals. *Neuropsychologia.* (2012) 50:2880–6. 10.1016/j.neuropsychologia.2012.08.011 22940426 PMC3473106

[4] Slot RER Verfaillie SCJ Overbeek JM Timmers T Wesselman LMP Teunissen CE Subjective cognitive impairment cohort (SCIENCe): study design and first results. *Alzheimers Res Ther.* (2018) 10:76. 10.1186/s13195-018-0390-y 30081935 PMC6080529

[5] Reisberg B Prichep L Mosconi L John ER Glodzik-Sobanska L Boksay I The pre-mild cognitive impairment, subjective cognitive impairment stage of Alzheimer’s disease. *Alzheimers Dement.* (2008) 4 (1 Suppl. 1):S98–108. 10.1016/j.jalz.2007.11.017 18632010

[6] St John P Montgomery P. Are cognitively intact seniors with subjective memory loss more likely to develop dementia? *Int J Geriatr Psychiatry.* (2002) 17:814–20. 10.1002/gps.559 12221654

[7] Wang L van Belle G Crane PK Kukull WA Bowen JD McCormick WC Subjective memory deterioration and future dementia in people aged 65 and older. *J Am Geriatr Soc.* (2004) 52:2045–51. 10.1111/j.1532-5415.2004.52568.x 15571540

[8] Jorm AF Christensen H Korten AE Jacomb PA Henderson AS. Memory complaints as a precursor of memory impairment in older people: a longitudinal analysis over 7-8 years. *Psychol Med.* (2001) 31:441–9. 10.1017/S0033291701003245 11305852

[9] Rönnlund M Sundström A Adolfsson R Nilsson LG. Subjective memory impairment in older adults predicts future dementia independent of baseline memory performance: evidence from the Betula prospective cohort study. *Alzheimers Dement.* (2015) 11:1385–92. 10.1016/j.jalz.2014.11.006 25667997

[10] Rakesh G Szabo ST Alexopoulos GS Zannas AS. Strategies for dementia prevention: latest evidence and implications. *Ther Adv Chronic Dis.* (2017) 8:121–36. 10.1177/2040622317712442 28815009 PMC5546647

[11] Rabin LA Smart CM Crane PK Amariglio RE Berman LM Boada M Subjective cognitive decline in older adults: an overview of self-report measures used across 19 international research studies. *J Alzheimers Dis.* (2015) 48 Suppl 1:S63–86. 26402085 10.3233/JAD-150154 PMC4617342

[12] Tandetnik C. *Ascertaining Subjective Cognitive Decline: A Comparison of Approaches and Evidence for Using an Age-Anchored Reference Group – PMC.* (2015). Available online at: https://www.ncbi.nlm.nih.gov/pmc/articles/PMC4598271/ (accessed September 2, 2022). 10.3233/JAD-150251 PMC4598271 26402092

[13] Hertzog C Hülür G Gerstorf D Pearman AM. Is subjective memory change in old age based on accurate monitoring of age-related memory change? Evidence from two longitudinal studies. *Psychol Aging.* (2018) 33:273–87. 10.1037/pag0000232 29658747

[14] Buckley R Saling MM Ames D Rowe CC Lautenschlager NT Macaulay SL Factors affecting subjective memory complaints in the AIBL aging study: biomarkers, memory, affect, and age. *Int Psychogeriatr.* (2013) 25:1307–15. 10.1017/S1041610213000665 23693133

[15] Sabatini S Ukoumunne OC Ballard C Collins R Anstey KJ Diehl M Cross-sectional association between objective cognitive performance and perceived age-related gains and losses in cognition. *Int Psychogeriatr.* (2021) 33:727–41. 10.1017/S1041610221000375 33849677

[16] Sabatini S Woods RT Ukoumunne OC Ballard C Collins R Clare L. Associations of subjective cognitive and memory decline with depression, anxiety, and two-year change in objectively-assessed global cognition and memory. *Neuropsychol Dev Cogn B Aging Neuropsychol Cogn*. (2022) 29:840–66. 10.1080/13825585.2021.1923634 33971790

[17] Scheltens NME Galindo-Garre F Pijnenburg YAL van der Vlies AE Smits LL Koene T The identification of cognitive subtypes in Alzheimer’s disease dementia using latent class analysis. *J Neurol Neurosurg Psychiatry.* (2016) 87:235–43. 10.1136/jnnp-2014-309582 25783437

[18] Pusswald G Moser D Gleiß A Auff E Dal-Bianco P Lehrner J Prevalence of mild cognitive impairment subtypes in patients attending a memory outpatient clinic-comparison of two modes of mild cognitive impairment classification. Results of the Vienna conversion to dementia study. *Alzheimers Dement.* (2012) 9:366–76. 10.1016/j.jalz.2011.12.009 23164551

[19] Kramer JH Jurik J Sha SJ Rankin KP Rosen HJ Johnson JK Distinctive neuropsychological patterns in frontotemporal dementia, semantic dementia, and Alzheimer disease. *Cogn Behav Neurol.* (2003) 16:211–8. 10.1097/00146965-200312000-00002 14665820

[20] Smits LL van Harten AC Pijnenburg YAL Koedam ELGE Bouwman FH Sistermans N Trajectories of cognitive decline in different types of dementia. *Psychol Med.* (2015) 45:1051–9. 10.1017/S0033291714002153 25229325

[21] Smart CM Krawitz A. The impact of subjective cognitive decline on Iowa gambling task performance. *Neuropsychology.* (2015) 29:971–87. 10.1037/neu0000204 26011116

[22] Smart CM Segalowitz SJ Mulligan BP MacDonald SWS. Attention capacity and self-report of subjective cognitive decline: a P3 ERP study. *Biol Psychol.* (2014) 103:144–51. 10.1016/j.biopsycho.2014.08.016 25204705

[23] Crane MK Bogner HR Brown GK Gallo JJ. The link between depressive symptoms, negative cognitive bias and memory complaints in older adults. *Aging Ment Health.* (2007) 11:708–15. 10.1080/13607860701368497 18074258 PMC2825049

[24] Hill NL Mogle J Wion R Munoz E DePasquale N Yevchak AM Subjective cognitive impairment and affective symptoms: a systematic review. *Gerontologist.* (2016) 56:e109–27. 10.1093/geront/gnw091 27342440 PMC5181393

[25] Mendonça MD Alves L Bugalho P. From subjective cognitive complaints to dementia: who is at risk?: a systematic review. *Am J Alzheimers Dis Other Demen.* (2016) 31:105–14. 10.1177/1533317515592331 26142292 PMC10852868

[26] Siebert JS Braun T Wahl HW. Change in attitudes toward aging: cognitive complaints matter more than objective performance. *Psychol Aging.* (2020) 35:357–68. 10.1037/pag0000451 32134302

[27] Yates JA Clare L Woods RT Matthews FE. Cognitive function and ageing study wales. subjective memory complaints are involved in the relationship between mood and mild cognitive impairment. *J Alzheimers Dis.* (2015) 48 (Suppl. 1):S115–23. 10.3233/JAD-150371 26402102

[28] Montejo P Montenegro M Fernandez MA Maestu F. Subjective memory complaints in the elderly: prevalence and influence of temporal orientation, depression and quality of life in a population-based study in the city of Madrid. *Aging Ment Health.* (2011) 15:85–96. 10.1080/13607863.2010.501062 20924824

[29] Zlatar ZZ Muniz M Galasko D Salmon DP. Subjective cognitive decline correlates with depression symptoms and not with concurrent objective cognition in a clinic-based sample of older adults. *J Gerontol B Psychol Sci Soc Sci.* (2018) 73:1198–202. 10.1093/geronb/gbw207 28329816 PMC6146771

[30] Schmidtke K Pohlmann S Metternich B. The syndrome of functional memory disorder: definition, etiology, and natural course. *Am J Geriatr Psychiatry.* (2008) 16:981–8. 10.1097/JGP.0b013e318187ddf9 19038897

[31] Lubitz AF Eid M Niedeggen M. Complainer Profile Identification (CPI): properties of a new questionnaire on subjective cognitive complaints. *Aging Neuropsychol Cogn.* (2018) 25:99–121. 10.1080/13825585.2016.1267325 27937808

[32] Anstey KJ. Optimizing cognitive development over the life course and preventing cognitive decline: introducing the cognitive health environment life course model (CHELM). *Int J Behav Dev.* (2014) 38:1–10. 10.1177/0165025413512255

[33] Heser K Tebarth F Wiese B Eisele M Bickel H Köhler M Age of major depression onset, depressive symptoms, and risk for subsequent dementia: results of the German study on ageing, cognition, and dementia in primary care patients (AgeCoDe). *Psychol Med.* (2013) 43:1597–610. 10.1017/S0033291712002449 23137390

[34] Pietrzak RH Maruff P Woodward M Fredrickson J Fredrickson A Krystal JH Mild worry symptoms predict decline in learning and memory in healthy older adults: a 2-year prospective cohort study. *Am J Geriatr Psychiatry.* (2012) 20:266–75. 10.1097/JGP.0b013e3182107e24 22354117 PMC3285262

[35] de Vito A Calamia M Greening S Roye S. The association of anxiety, depression, and worry symptoms on cognitive performance in older adults. *Neuropsychol Dev Cogn B Aging Neuropsychol Cogn.* (2019) 26:161–73. 10.1080/13825585.2017.1416057 29261012

[36] Abramson LY Metalsky GI Alloy LB. Hopelessness depression: a theory-based subtype of depression. *Psychol Rev.* (1989) 96:358–72. 10.1037/0033-295X.96.2.358

[37] Ho CS Feng L Fam J Mahendran R Kua EH Ng TP. Coexisting medical comorbidity and depression: multiplicative effects on health outcomes in older adults. *Int Psychogeriatr.* (2014) 26:1221–9. 10.1017/S1041610214000611 24735786

[38] Gallo JJ Bogner HR Morales KH Post EP Have TT Bruce ML. Depression, cardiovascular disease, diabetes, and two-year mortality among older, primary-care patients. *Am J Geriatr Psychiatry.* (2005) 13:748–55. 10.1097/00019442-200509000-00002 16166403 PMC2792894

[39] Bunce D Batterham PJ Christensen H Mackinnon AJ. Causal associations between depression symptoms and cognition in a community-based cohort of older adults. *Am J Geriatr Psychiatry.* (2014) 22:1583–91. 10.1016/j.jagp.2014.01.004 24502823

[40] Szanto K Dombrovski AY Sahakian BJ Mulsant BH Houck PR Reynolds CF Social emotion recognition, social functioning, and attempted suicide in late-life depression. *Am J Geriatr Psychiatry.* (2012) 20:257–65. 10.1097/JGP.0b013e31820eea0c 22354116 PMC3286029

[41] World Health Organization. *International Classification of Impairments, Disabilities, and Handicaps: A Manual of Classification Relating to the Consequences of Disease, Published in Accordance With Resolution WHA29.35 of the Twenty-Ninth World Health Assembly, 1976.* Geneva: World Health Organization (1980).

[42] Üstün TB Chatterji S Bickenbach J Kostanjsek N Schneider M. The international classification of functioning, disability and health: a new tool for understanding disability and health. *Disabil Rehabil.* (2003) 25:565–71. 10.1080/0963828031000137063 12959329

[43] Ustun TB Kostanjesek N Chatterji S Rehm J Organization WH. *Measuring Health and Disability: Manual for WHO Disability Assessment Schedule (WHODAS 2.0).* Geneva: World Health Organization (2010).

[44] Rotenberg S Leung C Quach H Anderson ND Dawson DR. Occupational performance issues in older adults with subjective cognitive decline. *Disabil Rehabil.* (2022) 44:4681–88. 10.1080/09638288.2021.1916626 33989108

[45] Cosentino S Devanand D Gurland B. A link between subjective perceptions of memory and physical function: implications for subjective cognitive decline. *J Alzheimers Dis.* (2018) 61:1387–98. 10.3233/JAD-170495 29376850 PMC6436538

[46] Federal Statistical Office. *Ticino.* (2019). Available online at: https://www.bfs.admin.ch/bfs/en/home/statistics/regional-statistics/regional-portraits-key-figures/cantons/ticino.html (accessed April 2022).

[47] Ibnidris A Piumatti G Carlevaro F Fadda M Magno F Magistro D Italian version of the short 10/66 dementia diagnostic schedule: a validation study. *BMJ Open.* (2021) 11:e045867. 10.1136/bmjopen-2020-045867 34193490 PMC8246379

[48] Stewart R Guerchet M Prince M. Development of a brief assessment and algorithm for ascertaining dementia in low-income and middle-income countries: the 10/66 short dementia diagnostic schedule. *BMJ Open.* (2016) 6:e010712. 10.1136/bmjopen-2015-010712 27225649 PMC4885443

[49] Rami L Mollica MA García-Sanchez C Saldaña J Sanchez B Sala I The subjective cognitive decline questionnaire (SCD-Q): a validation study. *J Alzheimers Dis.* (2014) 41:453–66. 10.3233/JAD-132027 24625794

[50] Beaton DE Bombardier C Guillemin F Ferraz MB. Guidelines for the process of cross-cultural adaptation of self-report measures. *Spine.* (2000) 25:3186–91. 10.1097/00007632-200012150-00014 11124735

[51] Ferreira D Falahati F Linden C Buckley RF Ellis KA Savage G A ‘disease severity index’ to identify individuals with subjective memory decline who will progress to mild cognitive impairment or dementia. *Sci Rep.* (2017) 7:44368. 10.1038/srep44368 28287184 PMC5347012

[52] van Oijen M de Jong FJ Hofman A Koudstaal PJ Breteler MMB. Subjective memory complaints, education, and risk of Alzheimer’s disease. *Alzheimers Dement.* (2007) 3:92–7. 10.1016/j.jalz.2007.01.011 19595922

[53] Hall KS Hendrie HC Britain HM Norton JA Rodgers DD Prince CS. The development of a dementia screeing interview in two distinct languages. *Int J Meth Psych Res.* (1993) 3:1–28.

[54] Fillenbaum GG van Belle G Morris JC Mohs RC Mirra SS Davis PC Consortium to establish a registry for Alzheimer’s disease (CERAD): the first twenty years. *Alzheimers Dement.* (2008) 4:96–109. 10.1016/j.jalz.2007.08.005 18631955 PMC2808763

[55] Gold LH. DSM-5 and the assessment of functioning: the world health organization disability assessment schedule 2.0 (WHODAS 2.0). *J Am Acad Psychiatry Law.* (2014) 42:9. 24986344

[56] Prince MJ Reischies F Beekman AT Fuhrer R Jonker C Kivela SL Development of the EURO-D scale–a European, Union initiative to compare symptoms of depression in 14 European centres. *Br J Psychiatry.* (1999) 174:330–8. 10.1192/bjp.174.4.330 10533552

[57] Liew TM. Depression, subjective cognitive decline, and the risk of neurocognitive disorders. *Alzheimers Res Ther.* (2019) 11:70. 10.1186/s13195-019-0527-7 31399132 PMC6689179

[58] Sousa RM Ferri CP Acosta D Albanese E Guerra M Huang Y Contribution of chronic diseases to disability in elderly people in countries with low and middle incomes: a 10/66 dementia research group population-based survey. *Lancet.* (2009) 374:1821–30. 10.1016/S0140-6736(09)61829-8 19944863 PMC2854331

[59] Hopper S Hammond NG Taler V Stinchcombe A. Biopsychosocial correlates of subjective cognitive decline and related worry in the Canadian longitudinal study on aging. *Gerontology.* (2022) 9:1–14. 10.1159/000524280 35533660 PMC9808637

[60] Cohen J. *Statistical Power Analysis for the Behavioral Sciences.* 2nd ed. New York, NY: Routledge (1988). p. 567.

[61] StataCorp. *Stata Statistical Software: Release 16.* College Station, TX: StataCorp (2017).

[62] Liu T Hardy SE editors. *Subjective Memory Complaints and Functional Status, Medicare Expenditure and Hospitalization.* Hoboken, NJ: Wiley-Blackwell (2013).

[63] Molinuevo JL Rabin LA Amariglio R Buckley R Dubois B Ellis KA Implementation of subjective cognitive decline criteria in research studies. *Alzheimers Dement.* (2017) 13:296–311. 10.1016/j.jalz.2016.09.012 27825022 PMC5344703

[64] Roehr S Riedel-Heller SG Kaduszkiewicz H Wagner M Fuchs A van der Leeden C Is function in instrumental activities of daily living a useful feature in predicting Alzheimer’s disease dementia in subjective cognitive decline? *Int J Geriatr Psychiatry.* (2019) 34:193–203. 10.1002/gps.5010 30353573

[65] Teng E Becker BW Woo E Knopman DS Cummings JL Lu PH. Utility of the functional activities questionnaire for distinguishing mild cognitive impairment from very mild Alzheimer disease. *Alzheimer Dis Assoc Disord.* (2010) 24:348–53. 20592580 10.1097/WAD.0b013e3181e2fc84 PMC2997338

[66] Sosa AL Albanese E Stephan BCM Dewey M Acosta D Ferri CP Prevalence, distribution, and impact of mild cognitive impairment in Latin America, China, and India: a 10/66 population-based study. *PLoS Med.* (2012) 9:e1001170. 10.1371/journal.pmed.1001170 22346736 PMC3274506

[67] Sachs-Ericsson N Joiner T Plant EA Blazer DG. The influence of depression on cognitive decline in community-dwelling elderly persons. *Am J Geriatr Psychiatry.* (2005) 13:402–8. 10.1097/00019442-200505000-00009 15879589

[68] Kessler EM Bowen CE Baer M Froelich L Wahl HW. Dementia worry: a psychological examination of an unexplored phenomenon. *Eur J Ageing.* (2012) 9:275–84. 10.1007/s10433-012-0242-8 28804427 PMC5549110

[69] Alexopoulos GS Meyers BS Young RC Mattis S Kakuma T. The course of geriatric depression with “reversible dementia”: a controlled study. *Am J Psychiatry.* (1993) 150:1693–9. 10.1176/ajp.150.11.1693 8105707

[70] Alexopoulos GS. Depression in the elderly. *Lancet.* (2005) 365:1961–70. 15936426 10.1016/S0140-6736(05)66665-2

[71] Roe C Xiong C Miller JP Morris JC. Education and Alzheimer disease without dementia: support for the cognitive reserve hypothesis. *Neurology.* (2007) 68:223–8. 10.1212/01.wnl.0000251303.50459.8a 17224578

[72] Mol MEM Ruiter RAC Verhey FRJ Dijkstra J Jolles J. A study into the psychosocial determinants of perceived forgetfulness: implications for future interventions. *Aging Ment Health.* (2008) 12:167–76. 10.1080/13607860801972503 18389396

[73] Alzheimer’s Disease International. *World Alzheimer Report 2019 Attitudes to Dementia.* London: Alzheimer’s Disease International (2019).

[74] Domènech-Abella J Mundó J Switsers L van Tilburg T Fernández D Aznar-Lou I. Social network size, loneliness, physical functioning and depressive symptoms among older adults: examining reciprocal associations in four waves of the longitudinal aging study Amsterdam (LASA). *Int J Geriatr Psychiatry.* (2021) 36:1541–9. 10.1002/gps.5560 33908639

