# Supplementary material for: The role of disability and depressive symptoms in the relation between objective cognitive performance and subjective cognitive decline
Source: Front Psychiatry. 2022 Nov 24;13:963703. doi: 10.3389/fpsyt.2022.963703 (PMC9729556; doi:10.3389/fpsyt.2022.963703)
Supplement: Supplementary file 1 [file Table_1.DOCX]

Supplementary Material

**Supplementary Table 1. Number and proportion of participants having selected health conditions.**

| **Health conditions in the study sample (n= 250)** | |
| --- | --- |
|  | Yes, n (%) |
| High blood pression | 119 (47.6) |
| Missing | 6 (2.4) |
| Heart problems | 73 (29.2) |
| Missing | 8 (3.2) |
| Cerebrovascular accident | 9 (3.6) |
| Missing | 6 (2.4) |
| Transient ischemic attack | 12 (4.8) |
| Missing | 7 (2.8) |
| Episode of loss of consciousness | 14 (5.6) |
| Missing | 6 (2.4) |
| Diabetes | 36 (14.4) |
| Missing | 7 (2.8) |
| Chronic bad chest | 24 (9.6) |
| Missing | 6 (2.4) |
| Arthritis | 106 (42.4) |
| Missing | 8 (3.2) |
| Dyspnea | 21 (8.4) |
| Missing | 8 (3.2) |
| Gastrointestinal problems | 38 (15.2) |
| Missing | 8 (3.2) |
